# Factors Influencing Patient Pathways for Receipt of Cancer Care at an NCI-Designated Comprehensive Cancer Center

**DOI:** 10.1371/journal.pone.0110649

**Published:** 2014-10-20

**Authors:** Elizabeth A. Gage-Bouchard, Elisa M. Rodriguez, Frances G. Saad-Harfouche, Austin Miller, Deborah O. Erwin

**Affiliations:** 1 Department of Community Health and Health Behavior, School of Public Health and Health Professions, University at Buffalo, Buffalo, NY, United States of America; 2 Cancer Prevention & Population Sciences, Roswell Park Cancer Institute, Buffalo, NY, United States of America; 3 Department of Cancer Control Prevention & Population Sciences, Roswell Park Cancer Institute, Buffalo, NY, United States of America; 4 Department of Biostatistics & Bioinformatics, Roswell Park Cancer Institute, Buffalo, NY, United States of America; Newcastle University, United Kingdom

## Abstract

**Background:**

Within the field of oncology, increasing access to high quality care has been identified as a priority to reduce cancer disparities. Previous research reveals that the facilities where patients receive their cancer care have implications for cancer outcomes. However, there is little understanding of how patients decide where to seek cancer care. This study examined the factors that shape patients’ pathways to seek their cancer care at a National Cancer Institute-designated comprehensive cancer center (NCI-CCC), and differences in these factors by race, income and education.

**Methods:**

In-depth interviews and survey questionnaires were administered to a random sample of 124 patients at one NCI-CCC in the Northeast US. In-depth interview data was first analyzed qualitatively to identify themes and patterns in patients’ pathways to receive their cancer care at an NCI-CCC. Logistic Regression was used to examine if these pathways varied by patient race, income, and education.

**Results:**

Two themes emerged: following the recommendation of a physician and following advice from social network members. Quantitative data analysis shows that patient pathways to care at an NCI-CCC varied by education and income. Patients with lower income and education most commonly sought their cancer care at an NCI-CCC due to the recommendation of a physician. Patients with higher income and education most commonly cited referral by a specialist physician or the advice of a social network member. There were no statistically significant differences in pathways to care by race.

**Conclusions:**

Our findings show that most patients relied on physician recommendations or advice from a social network member in deciding to seek their cancer care at an NCI-CCC. Due to the role of physicians in shaping patients’ pathways to the NCI-CCC, initiatives that strengthen partnerships between NCI-CCCs and community physicians who serve underserved communities may improve access to NCI-CCCs.

## Introduction

Disparities in the burden of cancer by socioeconomic position (SEP), race and ethnicity are well documented in the United States [1]–[3]. The relationships between socio-demographic characteristics and cancer outcomes are complex, and disparities have been documented across the disease continuum from stage at diagnosis, access to high-quality care, prognosis, and mortality. In this paper, we focus on understanding the factors that influence patient selection of where they receive their cancer care, and how these processes vary by race and SEP.

Within the field of oncology, a growing body of research reveals that the settings where patients receive cancer care has implications for patients’ outcomes [4]–[7]. Previous research shows that patients from low SEP and African American cancer patients are less likely to receive optimal treatment such as surgery and chemotherapy for many major cancers, and are less likely to receive their care at National Cancer Institute (NCI)-designated comprehensive cancer centers (NCI-CCC) [8]–[12]. Cancer patients treated at institutions offering clinical treatment studies and performing a high volume of specialized care and surgeries for cancer tend to have better outcomes and fewer complications than patients treated in community hospitals with less expertise in specialized procedures [6], [13]. Additionally, patients receiving care at an NCI-CCC have significantly lower odds of mortality [7],[14]–[17]. Research has also shown that outcome disparities between African American and white patients are reduced when patients receive their care at an NCI-CCC [5]. These previous findings have focused attention on increasing access to care at NCI-CCCs for minority and underserved patients, specifically through identification and reduction of barriers to care [4], [7]. To this end, greater understanding is needed about the factors that influence patient selection of where they receive their cancer care.

In response to the evidence that access to, and type of, hospital may play an important role in cancer outcome disparities, various practice and policy changes have been developed to increase access to high quality care [18]–[20]. For example, NIH’s Revitalization Act of 1993 requires the inclusion of minorities and women by investigators conducting clinical studies [21]. This requirement supports inclusion and representation of all groups in NIH funded research by encouraging equal access to all therapeutic advances. Despite such initiatives, efforts to increase patient diversity have had mixed success [18], [19], [22], and social scientists have identified need to address political and structural inequalities in the general health care system, rather than focus on what may be termed “recruitmentology”, or reductionist attempts to include underrepresented racial/ethnic groups [23], [24]. While the need to increase diversity in patient populations at NCI-CCCs has been identified as a priority, there is an incomplete understanding of the factors that influence patient selection of where they receive their cancer care. Understanding patients’ pathways to care, and how these pathways vary by race and socioeconomic position, will foster development of interventions that increase minority patients’ access to NCI-CCCs.

This study used a mixed-methods approach to examine the associations between patient sociodemographic characteristics and the factors that influenced patients’ cancer care-seeking pathways to one NCI-CCC. The NCI-CCC where data were collected accepts all patients regardless of insurance status or ability to pay for care, removing these potential barriers to care for cancer patients within the catchment area from which we drew our sample. Using data from in-depth qualitative interviews, we analyzed patients’ narratives of their decision-making processes to gain nuanced insight into patients’ experiences and roles in deciding where to receive their cancer care. To examine if these processes varied by race, income, and education we then quantitatively examined the association between demographic characteristics and the factors that shaped patients’ decisions to seek their cancer care at the NCI-CCC (e.g. physician recommendation, media advertising, and advice from social networks).

## Participants and Methods

### Participant Recruitment and Setting

This study used a mixed-methods design, which included in-depth interviews and survey questionnaires, administered to all participants during a single data collection session. The sampling frame was a list of all white and African American patients over the age of 18 treated at one NCI-CCC in the Northeast US between October 2008 and December 2009. This institution is a free-standing facility not directly linked to any other health care system or university hospital and does not incorporate any primary care services as part of the facility. Therefore, all referrals for cancer care rely upon community physicians practicing outside the NCI-CCC. Patients must have a cancer diagnosis or be eligible for one of the specific high-risk clinics to be referred to the NCI-CCC. This NCI-CCC accepts all patients regardless of insurance status if they are in-state residents and all insurance programs available in the region are accepted. These circumstances provide unique opportunities to evaluate patient initiated cancer care as there is no internal or existing health care system referral source.

Respondents’ race was extracted from cancer registry data. All race/ethnicity and language preferences are collected for the cancer registry by hand-written forms completed by patients at the time of admittance to the facility. Only respondents who self-identified as African American or white at the time of diagnosis were eligible for the study. Eligible patients were randomly selected and sent a letter introducing them to the study. Randomization was achieved by assigning identification numbers to the complete list of eligible patients and a random number table was used to identify the first 50 participants. After the first round of letters and contacts were made, this process was reiterated until the sample sizes were obtained. African American patients were over-sampled from the patient population to improve representation in the sample. The recruitment letter introduced the study to potential participants and invited them to contact the study team directly. Individuals who did not respond to the recruitment letter received follow-up telephone calls at two-week intervals. Potential respondents who could not be reached after three telephone attempts were considered refusals. To protect respondent confidentiality, no further data are publically available.

### Procedures

The Institutional Review Board (IRB) at the NCI-CCC approved the study, and all respondents completed a written informed consent. All study team members were social scientists that were not involved in participants’ clinical care. Study team members met respondents in a survey research center in a non-clinical building at the cancer center. If participants were not able to come to the cancer center, interviewers traveled to respondents’ homes or offered the opportunity to participate over the telephone. All data were collected in one session. Participants first completed an in-depth interview conducted by race-concordant study team members. The open-ended interview guide was created based upon factors identified in previous research examining disparities in quality of cancer care, access to differential settings for cancer care, and care outcomes [6], [8], [10], [27]–[30]. Interviews lasted between thirty minutes and two hours, and were structured to examine respondents’ social networks, past health care experiences, experiences of discrimination, their experience navigating the system of cancer care after their diagnosis, and how they made decisions regarding their cancer care. All interviews were audio-recorded and transcribed verbatim. After the interview, respondents were asked to complete a survey questionnaire that included the socio-demographic questionnaire created by the MacArthur Foundation Research Network on Socioeconomic Status and Health. Participants received $50 compensation after completing the interview and survey.

### Qualitative Data Analysis

A codebook was developed based upon the interview guide and free coding of the first ten interview transcripts. This process yielded 25 data-driven codes. To initially sort the data, four members of the research team reviewed and coded each interview transcript. The coded data was entered into the qualitative data analysis software package NVivo8 (QSR International, Victoria, Australia). A second round of coding focused on the factors that influenced patient selection of where they receive their cancer care. The four coders independently read and coded the data using the codebook. Attention was paid to code frequency, code co-occurrence, and the context and meaning of codes for groups of respondents [31]. Codes were then categorized, and themes identified [31]. After independent coding was complete, the four coders met to discuss the themes, and established consensus on the primary themes identified in the data.

### Quantitative Data Analysis

#### Measures

Demographic measures were captured using the MacArthur Foundation Research Network on Socioeconomic Status and Health socio-demographic questionnaire [25], [26]. Fourteen items on the questionnaire measure subjective social status, educational attainment, occupational status, income, and assets. To capture educational attainment, we asked respondents “What is the highest degree you earned?” Response options included: high school diploma or equivalency (GED), Associates degree (junior college), Bachelor’s degree, Master’s degree, Doctorate, Professional (MD, JD, DDS, etc.), other, and none of the above. Total household income was measured by asking “Which of these categories best describes your total combined family income for the past 12 months? This should include income (before taxes) from all sources, wages, rent from properties, social security, disability and/or veteran’s benefits, unemployment benefits, workman’s compensation, help from relatives (including child payments and alimony), and so on.” Response items included: Less than $5,000, $5,000 through $11,999, $12,000 through $15,999, $16,000 through $24,999, $25,000 through $34,999, $35,000 through $49,999, $50,000 through $74,999, $75,000 through $99,999, $100,000 and greater, Don’t know, and No response. The MacArthur scale creates both the income and education variables as ordinal variables, not continuous. To capture household size, we asked “How many people are currently living in your household, including yourself?” The total household income and household size variables were used to calculate the percent of the federal poverty line variable for each respondent.

For quantitative analysis, respondents’ descriptions of their experiences seeking cancer care were coded and entered into the data software package SPSS 16.0. The variable “primary factor motivating care at NCI-CCC” was constructed by coding the in-depth interview data into the following: (1) primary care physician recommendation, (2) specialist recommendation, (3) clinician at diagnosing hospital recommendation, (4) encouraged by social networks, (4) gained information through media, (5) and gained information through community organizations. Responses that did not fit within this coding scheme were coded “other.” If respondents reported multiple factors motivating their decision to seek their cancer care at the NCI-CCC the interviewer asked them to identify the most important reason, and this response was coded for statistical analysis. To examine the role physicians play in shaping patient decision-making about where to receive their cancer care, we constructed a dichotomous variable, “physician vs. self-referral”. As presented in Table 1, respondents who identified a primary care physician, specialist, or clinician at diagnosing hospital as suggesting patients seek their cancer care at the NCI-CCC were coded “physician recommendation.” Respondents who described receiving advice from social network members, gaining information through media, or gaining information through community organizations as being their primary motivator to seek their cancer care at the NCI-CCC were coded as “self-referral.” Respondents who reported specifically not-following initial referral by physician were also coded as “self-referral”.

**Table 1:** Coding of Factors Motivating Cancer Care Seeking at a NCI-Designated Comprehensive Cancer Center.

Theme	Codes
**Physician** **Recommendation**	Primary Care Physician recommendation
	Specialist Physician recommendation
	Physician at diagnosing hospital recommendation (i.e. Hospitalist)
**Self-referral**	
Encouraged by socialnetwork members	Family member or friend previously treated at cancer center
	Family member or friend works at cancer center
	Family member of friend suggested cancer center
Gained informationthrough media	Print, radio, or television news
	Marketing campaigns
	Internet
Gained informationthrough communityorganizations	Charity resources (i.e. American Cancer Society)
	Faith-based organizations

#### Statistical Analysis

We converted income data to percent of the US federal poverty line, accounting for the number of family members in the household. The variables degree earned and percent poverty line were dichotomized for ease of interpretation. Cut points were chosen based on theoretically meaningful points; that is, individuals with a bachelor’s degree or greater have different resources than those with less than a bachelor’s degree. Similarly, individuals at or below the poverty line also have different resources and challenges than those who have income above the poverty line. Respondent data was summarized using descriptive statistics and contingency tables. Associations in the contingency tables were tested with Fisher’s Exact test.

The full dataset contained responses from 124 participants, with 84 MD referrals and 40 Self-referrals. With this binary endpoint, the limiting sample size was about 40, suggesting that 3–5 covariates could be supported without biases from over fitting. Complete information in the final model was available for 94 responders. The primary outcome was Referral type = MD (vs Self). All models described below were fit using multivariable Logistic Regression.

In the full model, the Referral type outcome was described as a function of main effects and second-order interactions for income, percent poverty, age, education, sex, race, employment marital status, and home ownership. Age was included as a continuous covariate without transformation. The largest homogeneous class served as the reference for categorical factors.

The full model was reduced in two specification stages. Stage 1 relied on Bootstrap methods [32]. In 2500 bootstrap replicates of the original dataset, the full model was reduced by backward selection with a p-value retention threshold of 0.10, constrained to respect the interaction term hierarchy. This threshold was a reasonable trade-off between covariate retention and convergence of the maximization process. At the retention threshold of 0.10, 1834/2500 = 73.4 percent of the bootstrap models converged at the last selection step.

The stage 1 model contained the main effect terms retained in at least 60% of the Bootstrap reductions: income, race, age, sec, education and work status. In the final model, Percent poverty was also retained, as we believe it to be a better indicator of financial resources than income.

In stage 2, nine covariate subset models were compared. These models compared the effects of income and/or percent poverty while adjusting for education, race, age, sex and work status. These subset models were compared on the basis of the Hosmer-Lemeshow goodness of fit test, the Area Under the Receiver Operating Characteristic Curve (AUC) and Akaike Information Criterion (AIC). The Hosmer-Lemeshow test assessed model calibration, comparing observed response proportions within deciles of response probabilities predicted by the model. P-values less than 0.05 were interpreted as a lack of model fit. The AUC measures the model ability to discriminate between the two possible outcomes. AUCs greater than 0.8 generally indicate good discrimination. AUC = 0.50 suggests the model predictions are no better than random chance. AIC measures the relative quality of the statistical model by penalizing the maximized likelihood function value for the number of parameters included. Smaller AIC values are generally preferred.

The final multiple Logistic Regression model for the outcome Referral type = MD (vs Self) was fit using percent poverty, education and race as the predictors. This model had Hosmer-Lemeshow p-value = 0.43, AUC = 0.67 and AIC = 114.3. These model fit statistics compared well to the Bootstrap specification model (Hosmer-Lemeshow p-value = 0.77, AUC = 0.69, AIC = 121.2). Modeling analyses were done using SAS/STAT software, Version 9.4. Copyright 2012, SAS Institute Inc. SAS is a registered trademark of SAS Institute Inc., Cary, NC, USA.

P values less than 0.05 were considered statistically significant. Odds ratio estimates include 95% confidence limits to describe the plausible range of values for the true (unknown) population parameter as supported by the data. The significance levels of individual tests were not adjusted to control the overall Type I error rate.

## Results

### Participants

Six hundred and thirteen individuals were invited to participate in the study. Of these potential respondents, 112 were determined to be ineligible for the study due to being deceased or inaccurate contact information. Of the 511 remaining potential respondents (324 white, 187 African American), 124 participated in the study (74 white and 50 African American) (Table 2). The response rate was 24%. Overall, the sample included patients with 19 different sites of cancer, with the two most common cancers being prostate (22%) and breast cancer (21%). The largest difference by race was in breast cancer: 32% of the African American sample compared to 13% of the white sample had this diagnosis. The African American sample had significantly less educational attainment and household income than the white sample. Forty-nine percent of the white sample had a college degree or more, compared to twenty-six percent of the African American sample. Similarly, sixty-five percent of the white sample had a household income that placed them at 200% of the federal poverty line or more, compared to twenty-six percent of the African American sample. The majority of white respondents (84%) owned their home compared to forty-two percent of the African American sample.

**Table 2:** Sample Characteristics.

	White		AfricanAmerican		
	N	%	N	%	
**Age**					
18–39	1	1%	0	0%	*NS*
30–39	2	3%	1	2%	
40–49	9	12%	9	18%	
50–59	20	27%	15	30%	
60–64	11	15%	9	18%	
65+	31	42%	16	32%	
Total	74	100%	50	100%	
**Gender**					
Female	38	51%	30	60%	*NS*
Male	36	49%	20	40%	
Total	74	100%	50	100%	
**Employment**					
Working full-time	22	30%	12	24%	*P<.05*
Working part-time	9	12%	2	4%	
Unemployed	6	8%	3	6%	
Retired	29	39%	14	28%	
Disabled	8	11%	18	36%	
No Response	0	0%	1	2%	
Total	74	100%	50	100%	
**Household Income**					
Less than $5,000	0	0%	2	4%	*P<.05*
$5,000 to $11,999	3	4%	8	16%	
$12,000 to $15,999	3	4%	2	4%	
$16,000 to $24,999	8	11%	7	14%	
$25,000 to $34,999	8	11%	5	10%	
$35,000 to $49,999	9	12%	2	4%	
$50,000 to $74,999	9	12%	3	6%	
$75,000 to $99,999	10	14%	2	4%	
$100,000 and more	14	19%	2	4%	
Missing/no response	10	14%	17	34%	
Total	74	100%	50	100%	
**Percent of Federal Poverty Line**					
Below or at Federal Poverty Line	12	16%	20	40%	*P<.001*
200% of Federal Poverty Line or more	48	65%	13	26%	
Missing	14	19%	17	34%	
Total	74	100%	50	100%	
**Home Ownership**					
Own Home	62	84%	21	42%	*P<.001*
Rents Home	10	14%	20	40%	
Other	2	3%	4	8%	
No Response	0	0%	5	10%	
Total	74	100%	50	100%	
**Education**					
Some college or less	38	51%	34	68%	*P<.05*
College degree or more	36	49%	13	26%	
No Response	0	0%	3	6%	
Total	74	100%	50	100%	
**Marital Status**					
Married or living with partner	50	68%	15	30%	*P<.001*
Widowed	7	9%	7	14%	
Divorced/Separated	9	12%	10	20%	
Never married	8	11%	17	34%	
No Response	0	0%	1	2%	
Total	74	100%	50	100%	
**Insurance Status**					
Medicaid	4	5%	11	22%	*P = .002*
Medicare	2	3%	3	6%	
Medicaid and Medicare	1	1%	6	12%	
Medicaid plus private health insurance	1	1%	1	2%	
Medicare plus private insurance	25	34%	7	14%	
Private health insurance or HMO	39	53%	19	38%	
No insurance	1	1%	2	4%	
No Response	1	1%	1	2%	
Total	74	100%	50	100%	
**Site of Cancer**					
Prostate	13	18%	14	28%	
Breast	10	13%	16	32%	
Head & Neck(e.g. thyroid, esophagus, larynx)	14	19%	3	6%	
Hematologic/Bone Marrow	13	18%	6	12%	
Gynecologic(e.g., uterus, vulva, ovarian, cervix)	5	7%	4	8%	
Gastrointestinal(e.g., colon, stomach)	6	8%	4	8%	
Bronchus & Lung	3	4%	3	6%	
Kidney	3	4%	0	0%	
Neuro-oncology(i.e., meninges)	3	4%	0	0%	
Skin	1	1%	0	0%	
Sarcoma & Melanoma	2	3%	0	0%	
Other (unclear primary)	1	1%	0	0%	
Total	74	100%	50	100%	

Abbreviations: NS, Not Significant. P values based on Pearson Chi-Square test results.

Some Columns do not equal 100% due to rounding.

### Qualitative Themes

#### Following the Recommendation of a Physician

The examination of patients’ narratives describing their pathways to cancer care at an NCI-CCC revealed two overarching themes: following the recommendation of a physician and following advice from social network members. One respondent described following the advice of her Primary Care Provider (PCP), “After I found out I had breast cancer, I was referred to [the NCI-CCC] by my primary doctor” [Respondent 4]. Another respondent described how he made his decision in consultation with his PCP, “[After being diagnosed with prostate cancer] my doctor recommended that I either go through the surgery or the external beam radiation. I decided to go with the option of the external beam radiation and he recommended that I go to [the NCI-CCC]” [Respondent 2].

Other patients were immediately advised to go to the NCI-CCC when they received their cancer diagnosis from an emergency room or local hospital. One respondent explains, “The doctors over at [local hospital] told me that I need to go to [NCI-CCC]” [Respondent 1]. Finally, after receiving their cancer diagnosis some respondents requested a second opinion and the specialist advised them to go to the NCI-CCC. One patient described this process, “The biopsy came back positive and I requested a second opinion. [The physician] was very good and said to go to the [NCI-CCC]” [Respondent 115].

#### The Influence of Social Network Members

A second theme in the patient narratives was relying on their social networks as sources of information and advice about cancer care. Some patients discussed their cancer diagnosis with their colleagues, friends or family members, and relied on the advice of their network members when deciding where to seek their cancer care. One respondent describes, “I worked for [local politician]…and he was always impressed with [NCI-CCC] and he often said that’s the place to go” [Respondent 59]. Similar to this respondent, some patients relied on the impressions their network members held of the NCI-CCC, and followed the advice of these respected network members when deciding where to seek their cancer care. Another respondent explained, “I have some co-workers who have come to [NCI-CCC] and everyone had said how great it is” [Respondent 87].

Some patients had previous experience with the NCI-CCC when caring for a friend or family member who had cancer in the past. One respondent diagnosed with prostate cancer explained, “My brother-in-law had seen [physician at NCI-CCC], he had some prostate concerns and he liked [the physician]” [Respondent 95]. Another respondent explained, “My sister-in-law had bladder cancer and she was treated [at the NCI-CCC] and she was treated wonderfully. I figured why not go to the best place if you have cancer” [Respondent 103]. Other respondents had experience with the NCI-CCC through friends or family who were employees of the cancer center. One respondent explained “My best friend’s daughter works at [NCI-CCC] and I contacted her and she hooked me up with somebody right there and got me in very quickly” [Respondent 117]. Another respondent explains, “The more I thought about it [NCI-CCC] is in our backyard, and my sister-in-law had been a secretary for [physician at cancer center] and knew him very well and respected him highly” [Respondent 144].

Finally, some patients had social network members who helped them access information related to their cancer diagnosis, and this information led them to seek their care at an NCI-CCC. One patient explained “[My doctor] told me to look on the Internet. I told him I don’t have Internet service. He never did give me any information on it, so I went home and told my sister. She looked up the number [NCI-CCC] and called…they were very good about giving me information that I needed” [Respondent 39]. Like this patient, some respondents had individuals within their social networks who helped them connect with information, which aided their decision-making surrounding where to receive their cancer care.

### Quantitative Differences in Factors Influencing Pathways to Seek Care at an NCI-designated Comprehensive Cancer Center

As Table 3 shows, the largest percentages of white respondents accessed care at the NCI-CCC following a specialist recommendation (36.5%) or advice from social network members (25.7%). In contrast, the largest proportion of African American respondents sought cancer care at the NCI-CCC following the recommendation of a physician (e.g. hospitalist) during the diagnosis process. These differences were marginally statistically insignificant (p = 0.057).

**Table 3:** Factors Motivating Cancer Care Seeking at a NCI-Designated Comprehensive Cancer Center by Race, Education, and Income.

	PCP	Specialist	Hospitalist	SocialNetworks	Media	CommunityOrganizations	Other	
**Race**								
White	14(18.9%)	27(36.5%)	10(13.5%)	19(25.7%)	1(1.4%)	0(0%)	3(4.1%)	*P = .057*
AfricanAmerican	10(20.0%)	10(20.0%)	13(26.0%)	9(18.0%)	0(0%)	2(4.0%)	6(12.0%)	
**Educational** **Attainment**								
Some collegeor less	17(23.6%)	20(27.8%)	18(25.0%)	11(15.3%)	0(0%)	1(1.4%)	5(6.9%)	*P<.05*
College degreeor more	7(14.3%)	17(34.7%)	3(6.1%)	17(34.7%)	1(2.0%)	0(0%)	4(8.2%)	
**Percent Federal** **Poverty Line**								
Below or atFederal PovertyLine	6(18.8%)	8(25%)	10(31.3%)	3(9.4%)	0(0%)	1(3.1%)	4(12.5%)	*P<.05*
200% of FederalPoverty Line orMore	13(21.3%)	24(39.3%)	4(6.7%)	16(26.2%)	1(1.6%)	0(0%)	3(4.9%)	

Education was a statistically significant factor in determining respondents’ methods of accessing care at an NCI-CCC. Respondents who had some college or less most commonly sought their cancer care at the NCI-CCC based on a recommendation from their Primary Care Provider (PCP), a specialist, or a physician at the diagnosing hospital (23.6%, 27.8%, and 25.0% respectively). However, the two most common factors influencing the care-seeking process for respondents with a college degree or more were the recommendation of a specialist (34.7%) and advice from social network members (34.7%). Finally, respondents with a household income at or below the federal poverty line were most likely to seek care at the NCI-CCC due to a recommendation from a specialist or physician at the diagnosing hospital (25% and 31.3% respectively). Respondents who had a household income above the federal poverty line most commonly identified a recommendation from a specialist (39.3%) or advice from a social network member (26.2%) as influencing their decision to seek their cancer care at an NCI-CCC.

When examining the dichotomized variable “physician versus self-referral”, differences emerged by education. As table 4 shows, controlling for income and race, patients with a college degree or more were less likely to seek care at the NCI-CCC solely on a physician’s recommendation OR = 0.33 (95% CI: 0.12 to 0.91) compared to respondents with some college or less.

**Table 4:** Associations between Demographic Characteristics and Deciding to Seek Cancer Care at a NCI-Designated Comprehensive Cancer Center due to a Physician Recommendation versus Self-referral.

Characteristic	OR (95% CI)	
**Race**		
White	1.00	[Reference]
African American	0.38	(0.13–1.13)
**Education**		
Some College or Less	1.00	[Reference]
College Degree or More	0.33	(0.12–0.91)^s^
**Percent of Federal Poverty Line**		
At or below the federal poverty line	1.00	[Reference]
200% of federal poverty line or more	0.65	(0.21–2.06)

Abbreviations: OR, odds ratio; CI, 95% confidence interval.

s Significant at P<.05.

## Discussion

Recent research has highlighted the importance of the setting of cancer care on cancer outcomes in the United States [4], [7], [33]. However, there is little understanding of the processes surrounding patients’ decisions on where they receive cancer care and differences in these factors by race, income and education. Social capital (the benefits and challenges that accrue from participation in social networks and groups) [34], [35], provides a framework for understanding the factors that may shape decisions of where to receive cancer care [36]–[38]. Most patients in our sample made the decision to seek their cancer care at an NCI-CCC based upon a physician recommendation or the advice of a social network member, both highlighting the role of social relationships in shaping pathways to cancer care [39]. The factors that influenced respondents’ decisions surrounding where to receive their cancer care varied by patient SEP. Patients with higher income and education levels were able to seek multiple opinions and often relied on the recommendation of a specialist. Higher SEP patients may be more likely to access multiple forms of social capital [40] and therefore have the resources, experience and social contacts to actively seek the opinions of other professionals and members of their social networks when deciding where to receive their cancer care. Patients with lower income and education may be less likely to have these resources, and therefore, most commonly followed the recommendation of their primary care physician, specialist or hospital referral. Findings from previous research show that cancer patients who received the majority of their care from generalists rather than a specialist were less likely to attend an NCI-CCC [4]. The lower SEP patient narratives seldom reported patients seeking a clinical second opinion or other sources of information, even when prompted about these opportunities by the interviewer. Notably, very few patients in the entire sample reported decisions based upon media, which included Internet, or marketing information they had heard or received.

Our sample had a limited number of high SEP African American respondents, and there was a close association between race and income. This particular finding exemplifies and reaffirms the complexity of health disparities with respect to race, historical experiences of a particular group, and a group’s socioeconomic level [20]. The persistence of cancer health disparities is related to multiple factors and is often a mixture of factors depending on the cancer and/or group of interest. The finding that many patients from lower SEP environments are more dependent on clinical referrals from community physicians or hospitalists, and may not have the resources or experiences to investigate multiple clinical options, suggests a need for NCI-CCCs to evaluate their role and relationships within their local healthcare environments. Additionally, there may also be opportunities for policy and procedural changes within the local health systems to improve access to NCI-CCCs by examining the processes that shape generalist providers’ and emergent care facilities’ referrals to cancer care.

### Limitations

While these findings contribute insight to factors that shape patients’ pathways to receiving cancer care, some data limitations should be noted when interpreting our findings. While our mixed-methods design permits in-depth analysis of patients’ narratives impacting their care-seeking processes, our sample only includes patients who accessed care at one NCI-CCC. These processes may vary for patients in other geographic locations, especially in catchment areas where lack of health insurance may present a barrier to care at an NCI-CCC. Likewise, historical influences [20], individual institutional reputations, length of time in existence and physical location [4], [33] within a city or region may impact access. Future research that compares the experiences of these patients with patients who sought care in settings other than NCI-CCCs would provide further insight into the factors that shape patients’ care-seeking. Due to the small sample size, this sample does not permit examination of other factors, such as cancer site or prognosis, which likely play a role in patients’ decisions of where to receive their cancer care. An important direction for future research is to build upon these findings to examine how other patient, disease, and geographic characteristics shape patient pathways to cancer care. Similarly, future research should examine how insurance status, and type of health insurance, shape patient experiences deciding where to receive their cancer care.

### Conclusions and Implications for Practice

These results show that the majority of white (69%) and African American (66%) respondents relied on the referral of a physician seen during the time of diagnosis in deciding where to receive their cancer care. For African Americans, this was most often a physician at a community hospital (i.e., Hospitalist), and for whites, a specialist physician. These results highlight the importance of the referring physician’s relationship with local NCI-CCC in influencing where patients receive cancer care. In our sample, even patients who actively sought information about treatment options, obtained second opinions, and sought counsel from their social networks, relied significantly on the professional opinions of the physicians and specialists that were part of the diagnostic process. In this age of consumer-oriented medical care, and physician ownership of therapeutic and surgical centers, it is important to understand how, when, and why physicians refer patients to certain facilities after a cancer diagnosis. Future research should examine the perspectives, knowledge, and motivating factors of physicians, and variables that influence physicians’ decisions surrounding where to suggest patients receive their cancer care.

Understanding how patients seek and are directed into their cancer care provides us with better information to design and implement more effective outreach to underserved patients in addition to identifying specific areas for improvement in systems of care. These findings have significance for efforts aimed at increasing access to NCI-CCCs, and impacting health disparities in cancer care and outcomes. Our findings show that physicians at multiple levels serve an important function in increasing access to NCI-CCCs though their role in offering recommendations through the diagnosis process. Future directions for interventions should include opportunities for collaboration with safety-net providers and systems of community health care in an effort to build partnerships and capacity as a way to increase access to NCI-CCCs. Our findings also show that patients relied on connections to the NCI-CCC through their social network when deciding where to seek their cancer care. Important network ties included cancer center employees, current or former patients who had a positive experience at the cancer center, or network members who had a positive impression of the cancer center. These connections form a type of social capital that may impact cancer care-seeking, access, and ultimately disease outcomes. These findings suggest that increased diversity among cancer center employees and patients may positively impact future patients’ access and pathways to cancer care at NCI-CCCs.
